# Comparing writing style feature-based classification methods for estimating user reputations in social media

**DOI:** 10.1186/s40064-016-1841-1

**Published:** 2016-03-02

**Authors:** Jong Hwan Suh

**Affiliations:** Moon Soul Graduate School of Future Strategy, KAIST, 291 Daehak-ro, Yuseong-gu, Daejeon, 34141 Republic of Korea

**Keywords:** User reputation estimation, Social media, Writing style features, Classification techniques, Ensemble learning, Comparative studies

## Abstract

In recent years, the anonymous nature of the Internet has made it difficult to detect manipulated user reputations in social media, as well as to ensure the qualities of users and their posts. To deal with this, this study designs and examines an automatic approach that adopts writing style features to estimate user reputations in social media. Under varying ways of defining Good and Bad classes of user reputations based on the collected data, it evaluates the classification performance of the state-of-art methods: four writing style features, i.e. lexical, syntactic, structural, and content-specific, and eight classification techniques, i.e. four base learners—C4.5, Neural Network (NN), Support Vector Machine (SVM), and Naïve Bayes (NB)—and four Random Subspace (RS) ensemble methods based on the four base learners. When South Korea’s Web forum, Daum Agora, was selected as a test bed, the experimental results show that the configuration of the full feature set containing content-specific features and RS-SVM combining RS and SVM gives the best accuracy for classification if the test bed poster reputations are segmented strictly into Good and Bad classes by portfolio approach. Pairwise *t* tests on accuracy confirm two expectations coming from the literature reviews: first, the feature set adding content-specific features outperform the others; second, ensemble learning methods are more viable than base learners. Moreover, among the four ways on defining the classes of user reputations, i.e. like, dislike, sum, and portfolio, the results show that the portfolio approach gives the highest accuracy.

## Background

For the last decade, the development of the Internet and mobile devices has increased the popularity of social media (Sherchan et al. 2013; Beato et al. 2015). Social media varies in form and purpose, including blogs, e.g. Blogspot, microblogs, e.g. Twitter, discussion forums, e.g. Epinions, media sharing sites, e.g. YouTube, and social networks, e.g. Facebook (Kaplan and Haenlein 2010; Sun et al. 2015). People take advantage of social media as an electronic communication platform to share information, express their opinions, and construct their social networks, and furthermore to hear the voice of other users (Suh 2015; Sherchan et al. 2013; Li et al. 2013). Besides, information and opinion, shared and shaped through social media, influence individual views of society and incite on and offline political participations, e.g. South Korea candlelight protest of 2008 and United States Occupy Wall Street of 2011. Thus, now social media has become an open platform for political and social innovations (Suh 2015; de Zuniga 2012).

However, there are problems and challenging issues for social media to grow into a better online place for political and social innovations, as well as for trusty information and opinions sharing. First, the anonymous nature of the Internet makes it difficult to ensure the qualities of users and their posts in social media. If there is no online user feedback on an anonymous user’s posts, e.g. like and dislike, there is no way to find whether the anonymous user is good or bad before reading her/his post. This makes users in social media vulnerable to low quality posts and posters, offending and deceiving. Second, manipulations on user reputations arouse suspicion and mistrust on the online feedback system of social media in two ways: good quality posts and their users can be given low reputations maliciously by other users; some users can manipulate their own reputations to be high for certain reasons. However, identity changes, stemming from the anonymity of the Internet, lead to insufficient past reputation records on social media users, and make it hard to detect the manipulations on user reputations by the existing approaches, e.g. suggested in Lai et al. (2013).

To resolve the abovementioned problems and challenging issues, the user reputations in social media need to be estimated upon concrete user features. Moreover, previous works hint that writing styles of social media users can be such objective features (Koppel et al. 2009). Therefore, this paper proposes an automatic approach that adopts the writing styles of users as objective features for estimating user reputations in social media. Nevertheless, following research gaps are identified through the literature reviews: first, no study has been made on an automated classification of user reputations in social media by using writing style analysis; second, when a way of defining user reputations into Good and Bad classes is given, it is unclear which writing style feature and classification technique will be better for this study; third, there is no reference on how to define the classes of user reputations in social media for the better performance.

Therefore, this paper proposes a research framework to find out a better way in estimating user reputations in social media by using writing style features. To explain, first of all, the paper segments the test bed users into Good or Bad reputation classes by proposed four approaches: like, dislike, sum, and portfolio. Moreover, it extracts four writing style features, i.e. lexical (denoted by F1), syntactic (denoted by F2), semantic (denoted by F3), and content-specific (denoted by F4), to represent the reputations of the test bed users. Next, it evaluates the classification performance of 32 configurations, resulted by combining four feature sets, i.e. F1, F1 + F2, F1 + F2 + F3, and F1 + F2 + F3 + F4, and eight classification techniques, i.e. four base learners—C4.5, Neural Network (NN), Support Vector Machine (SVM), and Naïve Bayes (NB)—and four Random Subspace (RS) ensemble methods based on the four base learners, with respect to accuracy for a given way of defining the classes of the test bed user reputations. In addition, it statistically compares the classification performances of different feature sets and different classification techniques by conducting pairwise *t* tests.

To sum up the contribution of this paper, it is the first work to deal with the estimation of user reputations in social media by using writing style features. If a system is built based on the experimental results of this study, the system can remedy the abovementioned shortcomings of social media’s reputation system as follows. First, the user reputation estimation based on writing style features helps protect users from being exposed to bad users and their harmful posts in social media. Second, it contributes to establishing trust among social media users when sharing and searching information and opinions. Eventually, these help for social media to evolve into the trustworthy virtual place for political/social innovations and information/opinion sharing.

The rest of this paper is organized as follows. “Literature reviews” section briefly introduces and reviews the relevant literature. “Proposed research framework” section outlines the proposed research framework, and explains it in detail. Subsequently, “Experimental results and discussions” section demonstrates and discusses the experimental results of applying the suggested research framework to the Web forum of South Korea, Daum Agora, chosen as a test bed. “Results on comparative studies” section evaluates the results with statistical comparisons. Finally, “Conclusions” section concludes the paper with a reflection on limitations and further works.

## Literature reviews

### Online anonymity

Online anonymity represents the incapability of others to identify an individual in computer-mediated communication (CMC) (Christopherson 2007). The online anonymity takes many different forms, grouped into three different types: first, visual anonymity is the most common type wherein physical characteristics are hidden although other identifying information is known; second, pseudonymity refers to the case when people use avatars or usernames as indicators of their online identity; third, full anonymity is said to exist where users remain unknowable after interaction has concluded, and occurs in the absence of any long-term usernames (Christie and Dill 2016). In this paper, the term anonymity refers to pseudonymity and full anonymity.

Due to the online anonymity, digesting posts in social media sometimes requires a great deal of risk taking, like doing businesses online without any physical interaction (Enders et al. 2008). For example, cyber criminals abuse the anonymous nature in social media to conduct malicious activities such as phishing scams, identity theft, and harassment (Iqbal et al. 2013). Hence, to alleviate such risk taking in reading posts, this paper aims at the objective user reputation system of social media, which is effective even under the anonymous circumstances.

### Online user feedbacks and user reputations in social media

Social media users post their opinions regarding particular objects such as products, services, companies, people, and events (Lai et al. 2013; Shad Manaman et al. 2016). Online user feedback mechanisms play crucial roles in evaluating the qualities of posts and their users. The online user feedbacks in social media are intended to offer social control mechanism, allowing social translucence for improved accountability (Erickson and Kellogg 2000). Mainly there are two types of online user feedbacks in social media: recommendations and reputations (Li et al. 2013). First, recommendations help users identify posts and users that suit their needs or preferences. They are usually used to solve information overload problems (Li et al. 2013). Recommendation systems are classified into content-based filtering, collaborative filtering, or hybrid approaches (Sarwar et al. 2001; Jin et al. 2004; Li et al. 2005; Huang et al. 2004; Liu et al. 2014; Yang et al. 2014). Second, reputations are considered as a collective measure of trustworthiness based on the referrals or ratings of users in social media (Jøsang et al. 2007). Reputation systems let users rate other users, and the ratings help determine who to trust in certain environments where users have to interact among themselves in online settings (Agudo et al. 2010).

Particularly for the reputation systems, there are two categories of calculating trust scores between users as user reputations: feature-based and graph-based. First, the feature-based method is to compute the trust score of an user from past ratings on the user’s posts (O’Donovan and Smyth 2005). Second, the graph-based approach is to derive the trust values based on explicitly specified relations (e.g. friends) or trust relationships of the user (Golbeck 2005). Between the two, this study adopts the first method that measures reputations from past ratings on the user’s posts because the anonymity accompanies uncovered or scarce relationships regarding the user.

### Writing style features for characterizing user reputations in social media

According to systematic functional linguistic theory, a language has the textual dimension which individuals use to convey their ideas varying stylistic elements in their writings. The writing styles are influenced by education, gender, and vocabulary as well as subconscious factors described in the psycholinguistics works. The statistical analysis on such writing styles, a.k.a. authorship analysis, can discriminate authorship in social media (Abbasi et al. 2008a). Because Web-based channels such as e-mail, newsgroup, and chat rooms are relatively casual compared with formal publications, social media users are more likely to leave their own styles in their writings (Zheng et al. 2006). Hence, if the reputations of the social media users are characterized by their writing styles, authorship analysis can help resolve the problem of anonymity in the online communications of social media (Zhao et al. 2015; Iqbal et al. 2013). Previous works on writing style analysis in Table 1 also hint that writing styles extracted from posts can be objective features that characterize user reputations in social media. Actually, it is practical to use the writing style features for the anonymous users in social media because other features, e.g. their relationships as graph-based features, are not available in most cases. Nonetheless, to our best knowledge, there is no previous work made on classifying the reputations of social media users by using their writing styles.

**Table 1:** Previous works that used writing style features in social media

Previous works	Tasks	Research purpose	Test bed social media	Language	Features of writing styles				Techniques
					Lexical	Syntactic	Structural	Content-specific	
Abbasi and Chen (2005)	Authorship identification	Evaluating the linguistic features of Web messages and comparing them to known writing styles offers the intelligence community a tool for identifying patterns of terrorist communication	Web forum	English, Arabic	√	√	√	√	C4.5, SVM
Zheng et al. (2006)	Authorship identification	Examining writing style features and classification techniques to identify authorship of unknown online messages	Internet news group, Bulletin board system (BBS)	English, Chinese	√	√	√	√	C4.5, NN, SVM
Argamon et al. (2007)	Classification	Developing a new type of lexical feature for stylistic text classification, and demonstrating its usefulness in sentiment classification	Movie reviews	English	√			√	SVM
Abbasi and Chen (2008)	Authorship identification, similarity detection	Using writing style analysis techniques for identification and similarity detection of anonymous identities	eBay comments, Java forum	English	√	√	√	√	SVM, RS-SVM, PCA, Standard K-L transforms
Abbasi et al. (2008b)	Classification	Evaluating techniques that select writing style features for sentiment classification	Movie reviews, Web forum	English, Arabic	√	√	√	√	IG, GA, SVM weights, EWGA
Abbasi et al. (2008a)	Similarity detection	Evaluating writing style similarity detection techniques	Online feedback comments of eBay members	English	√	√	√	√	PCA, *n*-gram models, Markov models, Cross entropy, K–L similarity
Agichtein et al. (2008)	Classification	Automatic finding on high-quality content in a question/answering portal	Yahoo! Answer	English	√	√		√	C4.5, SVM
Koppel et al. (2009)	Classification	Comparing methods and features applied to authorship attribution problems representative of the range of classical attribution problems	Blog	English	√	√	√	√	NB, C4.5, Window, Bayesian regression, SVM
Huang et al. (2010)	Classification	Evaluating the effectiveness of user-generated text data in online video classification	Video-sharing Web site	English	√	√		√	NB, C4.5, SVM
Zhang et al. (2011)	Classification	Evaluating writing style features and classification techniques for online gender classification	Web forum	English	√	√	√	√	SVM
Benjamin and Hsinchun (2012)	Classification	Investigating relationship between hacker posting behaviors and reputation to identify potential cues for determining key actors	Hacker communities	English			√		Regression analysis
Iqbal et al. (2013)	Authorship identification, classification	Studying three typical authorship analysis problems encountered by cybercrime investigators: authorship identification with large training samples, authorship identification with small training samples, and authorship characterization for gender and location	Blog	English	√	√	√	√	Ensemble of Nested Dichotomies, C4.5, RBF Network, NB, BayesNet
Jiang et al. (2014)	Similarity detection	Using online writing style analysis to segment the forum participants by stakeholder groups, and partitions their messages into different time periods of major firm events to examine how important stakeholders evolve over time	Web forum	English	√	√	√	√	EM clustering
This study	Classification	Using writing style features as objective features to estimate the classes of user reputations in social media	Web forum	Korean, English	√	√	√	√	C4.5, NN, SVM, NB, RS-C4.5, RS-NN, RS-SVM, RS-NB

Writing style markers that are known as the most effective discriminators of authorship in social media are lexical, syntactic, structural, and content-specific writing style features. Here, lexical, syntactic, and structural writing style features are called the content-free writing style features (Zhang et al. 2011; Jiang et al. 2014). Among the four writing style features in social media, the content-specific writing style features are expected to outperform the other content-free writing style features for this study because of their two characteristics: first, they consist of important keywords and phrases, so they are more meaningful with high representative ability than the other writing style features (Zhang et al. 2011); second, they contain a much larger number of *n*-grams extracted from the collected social media data, and the large potential feature spaces are known to be effective for online text classification (Abbasi and Chen 2008). Despite, all the writing style features used in Jiang et al. (2014) are considered for this study because there is no previous work that has shown which writing style feature is more useful for this study that aims to estimate user reputations in social media.

### Classification techniques for writing style analysis

Three major types of writing style analysis tasks are identification, similarity detection, and classification (Zheng et al. 2006; Abbasi and Chen 2005). First, identification entails comparing anonymous texts against those belonging to identified entities, where the anonymous text is known to be written by one of those entities. Second, similarity detection task requires the comparison of anonymous texts against other anonymous texts in order to assess the degree of similarity. Third, classification is related to categorizing objects in regards to their properties, e.g. gender, by using their writing styles as features that represent the properties. This study belongs to the third category of classification techniques for writing style analysis.

For the classification task, this paper adopts the supervised techniques because they have been extensively studied due to their predominant classification performance (Zheng et al. 2006; Abbasi and Chen 2009). In general, supervised techniques for classification consist of two steps: first, the extraction of features from training data and their conversion to feature vectors; second, training of the classifier on the feature vectors and application of the classifier to unseen instances. Hence, feature construction and learning method selection are crucial for accurate classification.

Referring to the classification techniques of previous writing style analysis works summarized in Table 1, four main supervised techniques are adopted as base learners for this study. To explain, first, C4.5, an extension of the ID3 algorithm, is a decision-tree building algorithm developed by Quinlan (1986), and it adopts a divide-and-conquer strategy and an entropy measure for object classification. Its goal is to classify mixed objects into their associated classes based on the objects’ attribute values. Second, NN has been popular because of its unique learning capability (Widrow et al. 1994), and has achieved good performance in many different applications (Giles et al. 1998; Kim and Lewis 2000; Tolle et al. 2000). Third, SVM is a novel learning machine first introduced by Vapnik (1995), and is based on the structural risk minimization principle from computational learning theory. Because SVM can handle millions of inputs with good performance (Cristianini and Shawe-Taylor 2000; Joachims 2002), it was introduced to writing style analysis in many previous works (Argamon et al. 2003; Vel et al. 2001; Diederich et al. 2003). Fourth, based on Bayes Theorem (Barnard 1958), NB is a fairly simple probabilistic classification algorithm that uses strong independence assumptions regarding various features (Yang et al. 2002). It assumes that the presence of any feature is entirely independent of the presence of the other features, and allows building classification models efficiently.

Among the four base learners, SVM is a highly robust technique that has provided powerful classification capabilities for online authorship analysis. In head-to-head comparisons, SVM significantly outperformed other supervised learning methods such as NN and C4.5 (Zheng et al. 2006; Abbasi and Chen 2009). Similarly, SVM is expected to outperform the other base learners for this study. However, for writing style analysis, it is unclear which classification technique consistently performs better than others for a given problem in a given domain.

Moreover, for this uncertainty, it is not uncommon to conduct multiple learners and create an integrated classifier based on overall performance (Wang et al. 2014). Hence, in addition to the four base learners, this paper combines an ensemble learning method to each of the four base learners. Ensemble learning is a machine learning paradigm where multiple learners are trained to solve the same problem. In contrast to base learners that try to learn one hypothesis from the training data, ensemble learning methods try to conduct a set of hypotheses and combine them for use. In general, ensemble learning methods are divided into two categories: first, Boosting and Bagging are instance partitioning methods; second, feature partitioning methods include RS (Polikar 2006; Zhou 2012; Wang et al. 2014).

For this study, RS is selected as an ensemble learning method because it showed better accuracy than Boosting and Bagging in Wang et al. (2014). RS is an ensemble construction technique proposed by (Tin Kam 1998), and modifies the training dataset in the feature space. RS considers that, if one obtain better base learners in random spaces than in the original feature space, the combined decision of such base learners can be superior to a single classifier constructed on the original training dataset in the complete feature sets (Wang et al. 2014). Eventually, RS is combined with the four base learners selected for this study, and the resulted four multiple learner methods, i.e. RS-C4.5, RS-NN, RS-SVM, and RS-NB, are additionally adopted for this study. Considering the superiority of SVM to the other three base learners, RS-SVM is expected to outperform the other three ensemble learning methods.

## Proposed research framework

**Fig. 1:**
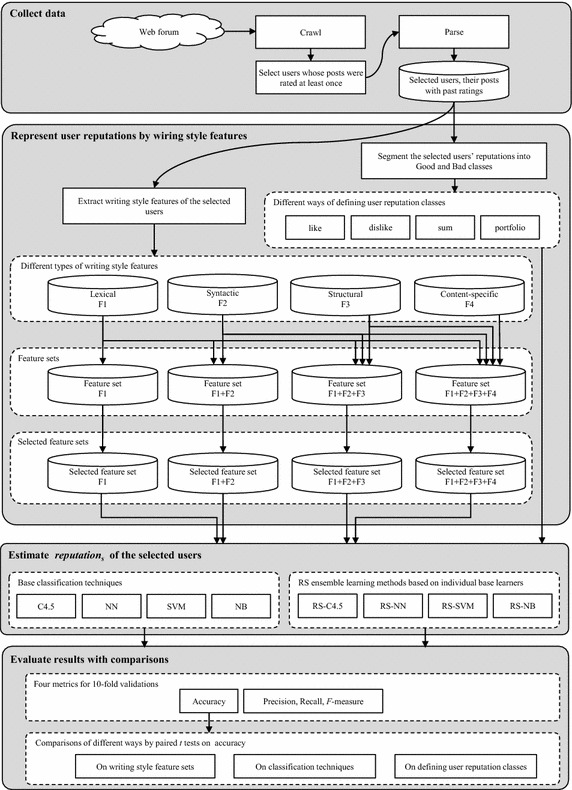
Research framework, proposed by this study

To design and examine an automatic approach that uses writing style features for estimating user reputations in social media, this study proposes a research framework as outlined in Fig. 1. The research framework answers below research questions.

- *RQ1* How does writing style features perform for estimating user reputations in social media?
- *RQ2* Which writing style features are the best at estimating user reputations by classification techniques in social media?
- *RQ3* Which classification technique is better suited at differentiating user reputations with writing style features in social media?
- *RQ4* Which method to define user reputation classes, i.e. Good and Bad, works better for estimating user reputations with writing style features in social media?

Ultimately, the research framework is intended for developing a system, which is capable of differentiating between Good and Bad reputation users by using stylistic tendencies inherent their writings in social media. In a nutshell, it consists of four steps: data collection, data representation, classification, and evaluation with comparisons. The following sub sections explain the details of each step in the research framework.

### Collect data

This study uses the Web forum for data collection because it is a major type of social media with a balanced nature of discussions among participants and a relatively broader range of topics (Zhang et al. 2011). The data collection from the Web forum has two steps, crawling and parsing. First, the developed Web crawler programs collect the online data from the Web forum as HTML pages. Then, users whose posts had been evaluated at least once by the others are selected, and the posts and their past ratings are parsed out for the selected users from the raw HTML pages, and are stored in a relational database.

### Represent user reputations by writing style features

In this step, writing style features are extracted as independent variables to represent the reputations of the collected and selected users from the Web forum. The class of a user reputation is obtained from her/his online user feedbacks by using different ways of defining user reputation classes. The details are explained in the following sub-sections.

#### Extract writing style features

This study generates different feature sets containing different types of writing style features. By doing so, we can compare and evaluate the performance of different writing style sets in estimating the classes of the selected users’ reputations. Table 2 lists the writing style features in social media, adopted for this study. In this paper, the different writing style features are denoted as follows: lexical features F1, syntactic features F2, structural features F3, and content-specific features F4. The writing style features of Table 2 are based on the prior studies in Table 1, mainly from Jiang et al. (2014). In addition, unlike the previous works, emotional writing style features are included to F1 for this study. The emoticon refers to graphic representations of facial expressions, which often follow utterances in written CMC, and are produced by ASCII symbols or by graphic symbols (Skovholt et al. 2014).

**Table 2:** Writing style features that are extracted from the posts of selected users for this study

	Type	Sub category	Writing style features (Korean and English)
F1	Lexical	Character-level	# of characters per a post Frequency of alphabetic characters, normalized by total number of characters Frequency of upper case characters, normalized by total number of characters (only English) Frequency of digit characters, normalized by total number of characters Frequency of white space characters, normalized by total number of characters Frequency of tab characters, normalized by total number of characters Frequency of letters, normalized by total number of alphabetic characters Frequency of special characters, normalized
		Word-level	# of words per a post Frequency of short words (length ≤3), normalized by total number of words Frequency of characters in words, normalized by total number of characters Average word length Average sentence length in terms of characters Average sentence length in terms of words Word length frequency (length ≤20), normalized by total number of words Frequency of emoticons per a post (e.g. :), :(, ㅠㅠ, -.-)
		Richness	Total different words, normalized by total number of words Hapax Legomena, normalized by total number of words Hapax Dislegomena, normalized by total number of words Yule’s K Simpson’s D Sichel’s S Brunet’s W Honore’s R
F2	Syntactic	–	Frequency of punctuations, normalized by total number of words Frequency of stop words, normalized by total number of words Frequency of POS *n*-grams (*n* = uni, bi, tri), normalized by total number of words Frequency of roots, normalized by total number of words
F3	Structural	–	# of sentences per a post Has greetings ∈ {0, 1} Has URLs ∈ {0, 1} Has quoted content including news ∈ {0, 1} Has e-mail as signature ∈ {0, 1} Has telephone number as signature ∈ {0, 1}
F4	Content-specific	Character-level	Character *n*-grams (*n* = uni, bi, tri), normalized by total number of characters
		Word-level	Word *n*-grams (*n* = uni, bi, tri), normalized by total number of words

As a result, the four types of writing style features, i.e. F1, F2, F3, F4, are obtained after feature extraction. Based on those different types of writing style features, four feature sets are constructed in an incremental way: feature set F1; feature set F1 + F2; feature set F1 + F2 + F3; feature set F1 + F2 + F3 + F4. This incremental order implies the evolutionary sequence of features (Zheng et al. 2006; Abbasi and Chen 2008; Zhang et al. 2011).

Next, for feature selection, information gain (IG) heuristic is adopted due to its reported effectiveness in previous online text classification. *IG* (*C*, *A*) measures the amount of entropy decrease on a class *C* when providing a feature *A* (Quinlan 1986; Shannon 1948; Zhang et al. 2011). The decreasing amount of entropy reflects the additional information gained by adding feature A, and higher values between 0 and 1 indicate more information gained by providing certain features (Zhang et al. 2011). In this study, writing style features with *IG* (*C*, *A*) > 0.0025 are selected by referring to previous related works (Yang and Pedersen 1997; Abbasi et al. 2008b; Zhang et al. 2011).

#### Segment the selected users’ reputations into Good and Bad classes

The selected Web forum users are segmented into Good and Bad reputation groups based on the ratings regarding their posts. In social media, there are generally two types of online user feedbacks: like and dislike, although there are various types of past ratings on their posts, e.g. helpfulness on reviews and reviewer rankings in Amazon.com, likes in Facebook, retweets in Twitter, ratings on sellers in eBay, ratings on answers in Q&A, etc. Hence, this paper proposes the four ways of defining social media users’ reputations into two classes: Good and Bad. The four approaches are named as segmenting type *s* = {like, dislike, sum, and portfolio}, and the reputation classes for users, reputation_*s*_, are respectively defined as

1 $$ reputation_{\text{like}} \left( {{\text{user}}_{i} } \right) = \left\{ {\begin{array}{ll} {\text{Good}} &\quad {{\text{if }}like\left( {user_{i} } \right) \ge m_{\text{like}} }  \\ {\text{Bad}} &\quad {\text{otherwise}} \\ \end{array} } \right\} \, , $$

where *like*(user_*i*_) is the number of likes that user_*i*_ obtained per a post in social media, and *m*_like_ is the average of *like*(user_*i*_) for *i* = 1, …, *N*.

2 $$ reputation_{\text{dislike}} \left( {{\text{user}}_{i} } \right) = \left\{ {\begin{array}{*{20}l} {\text{Good}} &\quad {{\text{ if }}dislike\left( {{\text{user}}_{i} } \right) < m_{\text{disike}} } \\ {\text{Bad}} &\quad {\text{otherwise}} \\ \end{array} } \right\} , $$

where *dislike*(user_*i*_) is the number of dislikes that user_*i*_ obtained per a post in social media, and *m*_dislike_ is the average of *dislike*(user_*i*_) for *i* = 1, …, *N*.

3 $$ reputation_{\text{sum}} \left( {{\text{user}}_{i} } \right) = \left\{ {\begin{array}{*{20}l} {\text{Good}} \hfill &\quad {{\text{if }}sum\left( {{\text{user}}_{i} } \right) \ge m_{\text{sum}} } \\ {\text{Bad}}  &\quad {\text{otherwise}} \\ \end{array} } \right\} , $$

where *sum*(user_*i*_) is equal to *like*(user_*i*_)—*dislike*(user_*i*_) and *m*_sum_ is the average of *sum*(user_*i*_) for *i* = 1, …, *N*.

4 $$ reputation_{\text{portfolio}} \left( {user_{i} } \right) = \left\{ {\begin{array}{*{20}l} {\text{Good}}  &\quad {{\text{if }}like\left( {user_{i} } \right) \ge m_{\text{like}} {\text{ and }}dislike\left( {user_{i} } \right) < m_{\text{dislike}} } \\ {\text{Bad}}  &\quad {\text{otherwise}}  \\ \end{array} } \right\} . $$

### Estimate *reputation*_*s*_ of the selected users

This paper adopts four base learners, i.e. C4.5, NN, SVM, and NB, commonly used for previous studies of writing styles in social media. Moreover, RS as an ensemble learning method is combined with the four base learners, resulting in RS-C4.5, RS-NN, RS-SVM, and RS-NB. In total, these eight classification techniques are used for this study. For an experiment, randomly 100 users for each reputation class are selected from the collected data, and a tenfold validation is performed to train a classifier and evaluate it. To implement the adopted eight classification techniques, the data mining toolkit WEKA (Waikato Environment for Knowledge Analysis) version 3.7.0 is used with all the default parameters because it is the most commonly used open-source toolkit with a collection of machine learning algorithms for solving data mining problems (Wang et al. 2014). In detail, the WEKA modules for algorithms used in this study are as follows: J48 module for C4.5, Multilayer Perceptron module for NN, SMO module for SVM, Naïve Bayes module for NB, and Random Subspace module for RS.

### Evaluate results with comparisons

To assess the performance of each feature set and each classification technique, this paper adopts the standard classification performance metrics. For the given segmenting type *s*, they are defined as

5 $$ accuracy_{s} = \frac{{|\{ {\text{users}}\;{\text{classified}}\;{\text{correctly}}\;{\text{either}}\;{\text{as}}\;reputation_{s} = {\text{Good}}\;{\text{or}}\;reputation_{s} = {\text{Bad}}\} |}}{{|\{ {\text{total}}\;{\text{users}}\;{\text{belonging}}\;{\text{either}}\;{\text{to}}\;reputation_{s} = {\text{Good}}\;{\text{or}}\;reputation_{s} = {\text{Bad}}\} |}} \, . $$

6 $$ precision_{s} (i) = \frac{{ | {\text{\{ users}}\;{\text{classified}}\;{\text{correctly}}\;{\text{as}}\;reputation_{s} = i\}|}}{{ | {\text{\{ users}}\;{\text{classified}}\;{\text{either}}\;{\text{correctly}}\;{\text{or}}\;{\text{falsely}}\;{\text{as}}\;reputation_{s} = i\} |}}\quad {\text{for }}i = {\text{Good}},\;{\text{Bad}} . $$

7 $$ recall_{s} (i) = \frac{{ |\{ {\text{users}}\;{\text{classified}}\;{\text{correctly}}\;{\text{as}}\;reputation_{s} = i\} |}}{{ |\{ {\text{users}}\;{\text{belonging}}\;{\text{to}}\;reputation_{s} \;{ = }\;i\} |}}\quad {\text{for }}i \, = {\text{ Good}},{\text{ Bad}} . $$

8 $$ F{ - }measure_{s} (i) = \frac{{2 \times precison_{s} (i) \times recall_{s} (i)}}{{(precision_{s} (i) + recall_{s} (i))}}\quad {\text{for }}i = {\text{Good}},\;{\text{Bad}} . $$

**Fig. 2:**
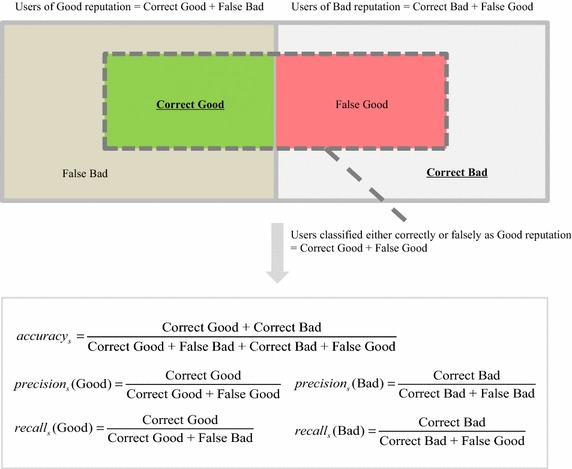
The illustration of accuracy, precision, and recall

To enhance understanding, Fig. 2 illustrates each metric of Eqs. (5)–(7). These four metrics have been widely used in information retrieval and text classification studies (Abbasi et al. 2008b; Li et al. 2008; Zhang et al. 2011). Among the four standard measures, accuracy assesses the overall classification correctness, while the others evaluate the correctness regarding each class. Therefore, this paper performs comparisons with respect to accuracy.

The comparisons are done by pairwise *t* tests because pairwise *t* test comparisons are the simplest kind of statistical tests and commonly used for comparing the performance of two algorithms (Derrac et al. 2011). To check whether the average difference in two approaches’ performance is significantly different from zero, this paper repeats the same experiments 50 times in two ways. First, to examine the effect of adding one feature set on accuracy for a certain classification technique, this paper conducts 24 (=three feature set comparisons × eight classification techniques) individual pairwise *t* tests. Second, to compare the performance of classification techniques on accuracy for a certain feature set, the paper conducts 64 (=24 + 24 + 16) individual pairwise *t* tests: 24 (=six technique comparisons × four feature sets) between base learners, i.e. Base versus Base, 24 (=six technique comparisons × four feature sets) between ensemble learning methods of RS, i.e. Ensemble versus Ensemble, and 16 (=four technique comparisons × four feature sets) between base learners and ensemble learning methods of RS, i.e. Base versus Ensemble.

## Experimental results and discussions

### Test bed: a Korean Web forum

This study targeted South Korea as a test bed country because South Korea has shown that social media can be used not only to exchange information and opinions, but also to organize the street protests and empower people to be active in the protests (Suh et al. 2010; Suh 2015). In particular, South Korea’s Web forum, called Daum Agora (http://agora.media.daum.net), was chosen as a test bed social media for three reasons: fist, Daum Agora is one of the most popular and anonymous Web forums in South Korea; second, it has been used from the early age of social media, i.e. the mid 2000 s. Hence, it contains a large-scale data with millions of posts and comments starting from August 2004; third, it is on a national scale so most of the main topics in South Korea are discussed through Daum Agora (Suh 2015). In this sense, Daum Agora proves sufficient and ideal for the Web forum of South Korea.

The web crawler program collected the posting data from Daum Agora, which had been generated for the past 5 years from 2007 to 2011, and were stored in the relational database for the experiments. In total, the online data on 2,565,918 posts from 91,968 users were collected. Among the collected users, users, of which posts had been evaluated at least once by the others, were selected for the experiments, and they are 22,131 users. Based on the collected data, the writing style features of 22,131 users are extracted.

Next, the online user feedbacks regarding the posts of the selected 22,1131 users were extracted from the collected data. In case of the test bed Daum Agora, there are two types of online user feedbacks: like and dislike. Based on these, the classes of the selected 22,131 users’ reputations were obtained by different segmenting types. As a result, Table 3 shows the number of users that belong to each user reputation class by different segmenting types. Moreover, from the selected 22,131 users, 100 users and their posts were randomly sampled for each class of user reputations in an experiment.

**Table 3:** The number of users that belong to each class of *reputation*_*s*_

Segmenting type *s*	Reputation_s_	*N*	Percent (%)
Like	Good	2355	10.65
	Bad	19,776	89.35
Dislike	Good	20,890	94.38
	Bad	1241	5.62
Sum	Good	2351	10.63
	Bad	19,780	89.37
Portfolio	Good	1909	8.63
	Bad	20,222	91.37

### Evaluations and discussions

Table 4 shows experimental results on accuracy for different writing feature sets and different classification techniques. To explain key findings, first, the feature set F1 + F2 + F3 + F4 gave the best accuracy for all the segmenting types except when segmenting type *s* = sum. On the other hand, RS-SVM gave the highest accuracy regardless of segmenting types. Second, among the 32 combinations, the feature set F1 + F2 + F3 + F4 and RS-SVM ranked the best, i.e. 94.50 %, in terms of accuracy for all the segmenting types. Likewise, the results in Table 4 indicate the superiority of the feature set F1 + F2 + F3 + F4 and RS-SVM. It is aligned with the paper’s expectations stated in the Literature Reviews section, and the possible reason is that their common advantage of handling with tens of thousands features made them have the best teamwork.

**Table 4:** Accuracy (%) for different feature sets and different classification techniques

Feature set	Base learners				Ensemble learning methods			
	C4.5	NN	SVM	NB	RS-C4.5	RS-NN	RS-SVM	RS-NB
(a) Segmenting type *s* = like								
F1	60.00	57.50	60.50	60.50	64.00	64.50	65.50	64.50
F1 + F2	57.00	54.50	58.50	53.00	68.00	68.50	68.50	59.50
F1 + F2 + F3	57.00	54.50	58.50	52.50	68.00	69.50	69.50	58.50
F1 + F2 + F3 + F4	62.00	66.00	69.00	65.50	77.00	79.00	*93.00*	84.50
(b) Segmenting type *s* = dislike								
F1	55.00	57.00	49.50	47.00	56.00	53.50	54.00	28.50
F1 + F2	58.50	59.00	57.50	24.00	64.00	67.00	65.50	44.50
F1 + F2 + F3	58.50	59.00	57.50	24.00	63.50	68.50	65.00	44.50
F1 + F2 + F3 + F4	68.00	71.50	74.00	65.50	86.00	93.50	*94.00*	85.50
(c) Segmenting type *s* = sum								
F1	67.50	66.00	60.50	64.50	72.50	68.00	63.00	63.50
F1 + F2	71.00	65.00	66.00	62.00	74.50	59.50	72.00	71.50
F1 + F2 + F3	71.00	66.50	66.00	62.00	72.00	59.00	70.50	71.50
F1 + F2 + F3 + F4	62.00	62.00	67.00	58.50	76.50	50.00	*82.50*	79.50
(d) Segmenting type *s* = portfolio								
F1	59.50	60.00	60.00	57.00	59.00	57.00	61.50	66.00
F1 + F2	65.50	62.50	63.50	60.00	71.50	65.00	77.50	74.00
F1 + F2 + F3	65.50	57.00	63.50	60.00	73.50	69.50	70.50	75.00
F1 + F2 + F3 + F4	64.50	70.50	72.50	76.50	80.00	87.00	***94.50***	88.50

The best result for each segmenting type is highlighted as italics, and the best result over all the segmenting types is additionally highlighted as bold italics

In addition, the best accuracies were identified respectively for all four segmenting types, and were compared to each other. For classification techniques, *reputation*_portfolio_ type gave the highest accuracy, i.e. 94.50 %, if the feature set F1 + F2 + F3 + F4 and the classification technique RS-SVM are used. On the other hand, *reputation*_sum_ type gave the lowest best accuracy, i.e. 82.50 %, if the feature set F1 + F2 + F3 and the classification technique RS-SVM are used. Thus, it is seen that the more accurate way of segmenting user reputations by portfolio approach contributed to its higher accuracy than the other segmenting ways. Whereas, *reputation*_sum_ type made it more difficult to classify user reputations, of which *like*(user_*i*_) and *dislike*(user_*i*_) are in a tense conflict.

Table 5 shows the evaluation results on precision, recall, and *F*-measure. To explain, the feature set F1 + F2 + F3 + F4 achieved the highest precisions: 98.88 % with RS-NN for *reputation*_dislike_ = Good, and 94.95 % with RS-SVM for *reputation*_portfolio_ = Bad. Next, the feature set F1 + F2 + F3 + F4 and RS-NN gave the highest recalls: 100 % for *reputation*_sum_ = Good, and 99.00 % for *reputation*_dislike_ = Bad. The highest *F*-measures were achieved by the feature set F1 + F2 + F3 + F4 in cooperation with RS-SVM when segmenting type *s* = portfolio, i.e. 94.53 % for *reputation*_portfolio_ = Good, and 94.47 % for *reputation*_portfolio_ = Bad. Putting together, these results show that the feature set F1 + F2 + F3 + F4 and ensemble learning methods gave the best precision, recall, and *F*-measure for both Good and Bad classes of user reputations.

**Table 5:** Performance measures (%) for different feature sets and different classification techniques

Feature set	Reputation_*s*_	Base learners											
		C4.5			NN			SVM			NB		
		Precision	Recall	*F-*measure	Precision	Recall	*F-*measure	Precision	Recall	*F-*measure	Precision	Recall	*F-*measure
(a) Segmenting type *s* = like													
F1	Good	63.89	46.00	53.49	56.76	63.00	59.72	64.38	47.00	54.34	64.38	47.00	54.34
	Bad	57.81	74.00	64.91	58.43	52.00	55.03	58.27	74.00	65.20	58.27	74.00	65.20
F1 + F2	Good	60.94	39.00	47.56	54.55	54.00	54.27	63.08	41.00	49.70	59.38	19.00	28.79
	Bad	55.15	75.00	63.56	54.46	55.00	54.73	56.30	76.00	64.68	51.79	87.00	64.93
F1 + F2 + F3	Good	60.94	39.00	47.56	54.64	53.00	53.81	63.08	41.00	49.70	58.06	18.00	27.48
	Bad	55.15	75.00	63.56	54.37	56.00	55.17	56.30	76.00	64.68	51.48	87.00	64.68
F1 + F2 + F3 + F4	Good	65.38	51.00	57.30	69.05	58.00	63.04	77.94	53.00	63.10	83.33	50.00	62.50
	Bad	59.84	73.00	65.77	63.79	74.00	68.52	64.39	85.00	73.28	64.29	81.00	71.68
(b) Segmenting type *s* = dislike													
F1	Good	58.62	34.00	43.04	67.50	27.00	38.57	49.33	37.00	42.29	63.41	26.00	36.88
	Bad	53.52	76.00	62.81	54.38	87.00	66.92	49.60	62.00	55.11	53.97	68.00	60.18
F1 + F2	Good	59.34	54.00	56.54	64.06	41.00	50.00	59.74	46.00	51.98	64.58	31.00	41.89
	Bad	57.80	63.00	60.29	56.62	77.00	65.25	56.10	69.00	61.88	56.67	17.00	26.15
F1 + F2 + F3	Good	59.34	54.00	56.54	64.06	41.00	50.00	59.74	46.00	51.98	64.58	31.00	41.89
	Bad	57.80	63.00	60.29	56.62	77.00	65.25	56.10	69.00	61.88	56.67	17.00	26.15
F1 + F2 + F3 + F4	Good	70.00	63.00	66.32	73.63	67.00	70.16	77.91	67.00	72.04	73.63	67.00	70.16
	Bad	66.36	73.00	69.52	69.72	76.00	72.73	71.05	81.00	75.70	77.11	64.00	69.95
(c) Segmenting type *s* = sum													
F1	Good	65.33	49.00	56.00	57.61	53.00	55.21	61.64	45.00	52.02	95.24	20.00	33.06
	Bad	59.20	74.00	65.78	56.48	61.00	58.65	56.69	72.00	63.44	55.06	*98.00*	70.50
F1 + F2	Good	64.71	55.00	59.46	58.95	56.00	57.44	69.14	56.00	61.88	75.47	40.00	52.29
	Bad	60.87	70.00	65.12	58.10	61.00	59.51	63.03	75.00	68.49	59.18	87.00	70.45
F1 + F2 + F3	Good	64.71	55.00	59.46	60.64	57.00	58.76	69.14	56.00	61.88	75.47	40.00	52.29
	Bad	60.87	70.00	65.12	59.43	63.00	61.17	63.03	75.00	68.49	59.18	87.00	70.45
F1 + F2 + F3 + F4	Good	69.12	47.00	55.95	58.62	51.00	54.55	70.11	61.00	65.24	75.68	56.00	64.37
	Bad	59.85	79.00	68.10	56.64	64.00	60.09	65.49	74.00	69.48	65.08	82.00	72.57
(d) Segmenting type *s* = portfolio													
F1	Good	60.00	57.00	58.46	59.62	62.00	60.78	61.11	55.00	57.89	54.93	78.00	64.46
	Bad	59.05	62.00	60.49	60.42	58.00	59.18	59.09	65.00	61.90	62.07	36.00	45.57
F1 + F2	Good	65.66	65.00	65.33	62.63	62.00	62.31	63.37	64.00	63.68	56.94	82.00	67.21
	Bad	65.35	66.00	65.67	62.38	63.00	62.69	63.64	63.00	63.32	67.86	38.00	48.72
F1 + F2 + F3	Good	65.66	65.00	65.33	56.03	65.00	60.19	63.37	64.00	63.68	56.94	82.00	67.21
	Bad	65.35	66.00	65.67	58.33	49.00	53.26	63.64	63.00	63.32	67.86	38.00	48.72
F1 + F2 + F3 + F4	Good	62.83	71.00	66.67	69.16	74.00	71.50	69.91	79.00	74.18	76.77	76.00	76.38
	Bad	66.67	58.00	62.03	72.04	67.00	69.43	75.86	66.00	70.59	76.24	77.00	76.62

Feature set	Reputation_*s*_	Ensemble learning methods											
		RS-C4.5			RS-NN			RS-SVM			RS-NB		
		Precision	Recall	*F-*measure	Precision	Recall	*F-*measure	Precision	Recall	*F-*measure	Precision	Recall	*F-*measure
(a) Segmenting type *s* = like													
F1	Good	66.28	57.00	61.29	64.36	65.00	64.68	67.42	60.00	63.49	66.67	58.00	62.03
	Bad	62.28	71.00	66.36	64.65	64.00	64.32	63.96	71.00	67.30	62.83	71.00	66.67
F1 + F2	Good	70.45	62.00	65.96	71.76	61.00	65.95	71.26	62.00	66.31	88.00	22.00	35.20
	Bad	66.07	74.00	69.81	66.09	76.00	70.70	66.37	75.00	70.42	55.43	*97.00*	70.55
F1 + F2 + F3	Good	70.45	62.00	65.96	71.43	65.00	68.06	71.91	64.00	67.72	86.96	20.00	32.52
	Bad	66.07	74.00	69.81	67.89	74.00	70.81	67.57	75.00	71.09	54.80	*97.00*	70.04
F1 + F2 + F3 + F4	Good	81.40	70.00	75.27	83.72	72.00	77.42	*96.74*	*89.00*	*92.71*	96.34	79.00	86.81
	Bad	73.68	84.00	78.50	75.44	86.00	80.37	*89.81*	*97.00*	*93.27*	82.57	90.00	86.12
(b) Segmenting type *s* = dislike													
F1	Good	54.76	69.00	61.06	52.26	81.00	63.53	52.25	93.00	66.91	71.88	23.00	34.85
	Bad	58.11	43.00	49.43	57.78	26.00	35.86	68.18	15.00	24.59	60.71	34.00	43.59
F1 + F2	Good	62.96	68.00	65.38	63.93	78.00	70.27	61.83	81.00	70.13	71.62	53.00	60.92
	Bad	65.22	60.00	62.50	71.79	56.00	62.92	72.46	50.00	59.17	87.80	36.00	51.06
F1 + F2 + F3	Good	63.11	65.00	64.04	66.09	76.00	70.70	61.54	80.00	69.57	71.62	53.00	60.92
	Bad	63.92	62.00	62.94	71.76	61.00	65.95	71.43	50.00	58.82	87.80	36.00	51.06
F1 + F2 + F3 + F4	Good	88.30	83.00	85.57	***98.88***	88.00	93.12	94.90	*93.00*	*93.94*	86.79	92.00	89.32
	Bad	83.96	89.00	86.41	89.19	***99.00***	93.84	93.14	95.00	*94.06*	***100.00***	79.00	88.27
(c) Segmenting type *s* = sum													
F1	Good	73.68	70.00	71.79	69.15	65.00	67.01	66.67	52.00	58.43	82.93	34.00	48.23
	Bad	71.43	75.00	73.17	66.98	71.00	68.93	60.66	74.00	66.67	58.49	93.00	71.81
F1 + F2	Good	78.16	68.00	72.73	63.77	44.00	52.07	73.91	68.00	70.83	77.22	61.00	68.16
	Bad	71.68	81.00	76.06	57.25	75.00	64.94	70.37	76.00	73.08	67.77	82.00	74.21
F1 + F2 + F3	Good	75.58	65.00	69.89	64.06	41.00	50.00	73.03	65.00	68.78	77.22	61.00	68.16
	Bad	69.30	79.00	73.83	56.62	77.00	65.25	68.47	76.00	72.04	67.77	82.00	74.21
F1 + F2 + F3 + F4	Good	82.72	67.00	74.03	50.00	***100.00***	66.67	81.55	84.00	*82.76*	*83.87*	78.00	80.83
	Bad	72.27	86.00	78.54	0.00	0.00	0.00	*83.51*	81.00	*82.23*	80.20	81.00	80.60
(d) Segmenting type *s* = portfolio													
F1	Good	58.82	60.00	59.41	57.29	55.00	56.12	60.95	64.00	62.44	65.09	69.00	66.99
	Bad	59.18	58.00	58.59	56.73	59.00	57.84	62.11	59.00	60.51	67.02	63.00	64.95
F1 + F2	Good	70.09	75.00	72.46	64.15	68.00	66.02	76.19	80.00	78.05	71.43	80.00	75.47
	Bad	73.12	68.00	70.47	65.96	62.00	63.92	78.95	75.00	76.92	77.27	68.00	72.34
F1 + F2 + F3	Good	71.56	78.00	74.64	69.70	69.00	69.35	71.13	69.00	70.05	71.93	82.00	76.64
	Bad	75.82	69.00	72.25	69.31	70.00	69.65	69.90	72.00	70.94	79.07	68.00	73.12
F1 + F2 + F3 + F4	Good	77.78	84.00	80.77	84.26	91.00	87.50	*94.06*	*95.00*	***94.53***	85.98	92.00	88.89
	Bad	82.61	76.00	79.17	90.22	83.00	86.46	*94.95*	*94.00*	***94.47***	91.40	85.00	88.08

For each segmenting type, the best results with respect to the three performance measures are highlighted in italics, and the best precision, recall, and *F*-measure over all 128(=4 feature types × 8 classification techniques × 4 segmenting types) are additionally highlighted as bold italics

## Results on comparative studies

### On comparisons of different feature sets

Table 6 shows the results of the pairwise *t* tests, conducted to examine the effect of different feature sets on accuracy for a certain classification technique. It reveals that, regardless of segmenting types, adding one type of writing style features improved most of classification accuracies except adding the structural features F3. The insignificant effect of adding F3 is because its size is small so its representation capability is smaller than adding the other features.

**Table 6:** Pairwise *t* tests on accuracy for different feature sets

	Base learners							
	C4.5		NN		SVM		NB	
	*t*	*p*	*t*	*p*	*t*	*p*	*t*	*p*
(a) Segmenting type *s* = like								
F1 < F1 + F2	*3.1271*	*0.0038*	*3.9988*	*0.0004*	*8.3702*	*0.0000*	*3.6306*	*0.0010*
F1 + F2 < F1 + F2 + F3	0.0266	0.9790	−*2.7879*	*0.0093*	0.0410	0.9676	0.0000	1.0000
F1 + F2 + F3 < F1 + F2 + F3 + F4	*5.9653*	*0.0000*	*11.9349*	*0.0000*	*9.8836*	*0.0000*	*28.3710*	*0.0000*
(b) Segmenting type *s* = dislike								
F1 < F1 + F2	1.1035	0.2744	0.7982	0.4280	*2.3112*	*0.0245*	−*11.0785*	*0.0000*
F1 + F2 < F1 + F2 + F3	0.0000	1.0000	0.0369	0.9707	0.0000	1.0000	0.0000	1.0000
F1 + F2 + F3 < F1 + F2 + F3 + F4	*3.2182*	*0.0021*	*4.8950*	*0.0000*	*5.7765*	*0.0000*	*16.0591*	*0.0000*
(c) Segmenting type *s* = sum								
F1 < F1 + F2	−0.5602	0.5775	1.7311	0.0888	*5.8095*	*0.0000*	−1.1181	0.2684
F1 + F2 < F1 + F2 + F3	−0.0182	0.9855	0.0488	0.9612	−0.0824	0.9346	0.0000	1.0000
F1 + F2 + F3 < F1 + F2 + F3 + F4	−*9.4704*	*0.0000*	−*9.2471*	*0.0000*	−0.5146	0.6088	−*2.9386*	*0.0058*
(d) Segmenting type *s* = portfolio								
F1 < F1 + F2	*3.1271*	*0.0038*	*3.9988*	*0.0004*	*8.3702*	*0.0000*	*3.6306*	*0.0010*
F1 + F2 < F1 + F2 + F3	0.0266	0.9790	−*2.7879*	*0.0093*	0.0410	0.9676	0.0000	1.0000
F1 + F2 + F3 < F1 + F2 + F3 + F4	*5.9653*	*0.0000*	*11.9349*	*0.0000*	*9.8836*	*0.0000*	*28.3710*	*0.0000*

	Ensemble learning methods							
	RS-C4.5		RS-NN		RS-SVM		RS-NB	
	*t*	*p*	*t*	*p*	*t*	*p*	*t*	*p*
(a) Segmenting type *s* = like								
F1 < F1 + F2	*11.8420*	*0.0000*	*11.7699*	*0.0000*	*18.7674*	*0.0000*	*17.8236*	*0.0000*
F1 + F2 < F1 + F2 + F3	0.9752	0.3370	0.4320	0.6687	−*3.4808*	*0.0015*	0.3547	0.7252
F1 + F2 + F3 < F1 + F2 + F3 + F4	*7.2221*	*0.0000*	*27.5745*	*0.0000*	*30.4662*	*0.0000*	*25.8677*	*0.0000*
(b) Segmenting type *s* = dislike								
F1 < F1 + F2	*2.3273*	*0.0235*	*3.6135*	*0.0006*	*3.8520*	*0.0003*	*7.6427*	*0.0000*
F1 + F2 < F1 + F2 + F3	−0.0326	0.9741	0.4753	0.6363	0.0316	0.9749	0.0000	1.0000
F1 + F2 + F3 < F1 + F2 + F3 + F4	*5.1154*	*0.0000*	*5.9364*	*0.0000*	*6.7400*	*0.0000*	*12.4236*	*0.0000*
(c) Segmenting type *s* = sum								
F1 < F1 + F2	*5.7001*	*0.0000*	−*4.7825*	*0.0000*	*9.0012*	*0.0000*	*27.3577*	*0.0000*
F1 + F2 < F1 + F2 + F3	−0.8536	0.3970	0.2574	0.7978	0.2793	0.7810	0.0000	1.0000
F1 + F2 + F3 < F1 + F2 + F3 + F4	1.1808	0.2426	−*15.1418*	*0.0000*	*9.2964*	*0.0000*	*20.1199*	*0.0000*
(d) Segmenting type *s* = portfolio								
F1 < F1 + F2	*11.8420*	*0.0000*	*11.7699*	*0.0000*	*18.7674*	*0.0000*	*17.8236*	*0.0000*
F1 + F2 < F1 + F2 + F3	0.9752	0.3370	0.4320	0.6687	−*3.4808*	*0.0015*	0.3547	0.7252
F1 + F2 + F3 < F1 + F2 + F3 + F4	*7.2221*	*0.0000*	*27.5745*	*0.0000*	*30.4662*	*0.0000*	*25.8677*	*0.0000*

The results are *t* and *p* values of the *t* tests for feature set comparisons, and the results more than 5 % of significance level are highlighted in italics

Moreover, the feature set F1 + F2 + F3 + F4 gave the best results for all eight classification techniques, regardless of the segmenting type. This suggests that the four feature sets provide important complementary and discriminatory potential if they are exploited by incorporating them in unison. Thus, a large set of rich writing style features are beneficial for automated classification on the reputations of social media users. Especially, it shows adding the content-specific writing style features F4 contributes to the best accuracy as expected in the Literature Reviews section. It indicates that keywords and phrases on certain topics are more important grounds to judge users than the other content-free writing style features in social media.

### On comparisons of different classification techniques

Table 7 shows the results of the pairwise *t* tests, performed to investigate the effect of different classification techniques on accuracy for a specific feature set. For a given segmenting type *s*, classification techniques were compared in three parts: Base versus Base, Ensemble versus Ensemble, and Base versus Ensemble. In Table 7, it was observed that the ranks of all eight classification techniques are different according to the selected feature set.

**Table 7:** Pairwise *t* tests on accuracy for different classification techniques

	F1		F1 + F2		F1 + F2 + F3		F1 + F2 + F3 + F4	
	*t*	*p*	*t*	*p*	*t*	*p*	*t*	*p*
(a) Segmenting type *s* = like								
Base versus Base								
C4.5 < NN	0.1168	0.9074	−0.2642	0.7926	−0.2391	0.8119	*2.0894*	*0.0411*
C4.5 < SVM	0.4121	0.6818	−0.1558	0.8768	−0.1733	0.8630	1.9855	0.0519
C4.5 < NB	0.4411	0.6608	−1.6644	0.1015	−1.7821	0.0800	0.7397	0.4625
NN < SVM	0.2948	0.7692	0.1085	0.9140	0.0661	0.9475	−0.0980	0.9222
NN < NB	0.3237	0.7473	−1.3992	0.1671	−1.5415	0.1287	−1.3530	0.1813
SVM < NB	0.0287	0.9772	−1.5085	0.1369	−1.6103	0.1128	−1.2512	0.2159
Ensemble versus Ensemble								
RS-C4.5 < RS-NN	0.0985	0.9219	0.0305	0.9758	0.0407	0.9677	0.8816	0.3816
RS-C4.5 < RS-SVM	0.2781	0.7819	−0.0970	0.9231	−0.0561	0.9554	*3.3050*	*0.0017*
RS-C4.5 < RS-NB	0.3815	0.7043	−*3.1268*	*0.0028*	−*3.1920*	*0.0023*	1.5449	0.1278
RS-NN < RS-SVM	0.1791	0.8585	−0.1273	0.8992	−0.0967	0.9233	*2.4291*	*0.0183*
RS-NN < RS-NB	0.2820	0.7790	−*3.1492*	*0.0026*	−*3.2248*	*0.0021*	0.6608	0.5113
RS-SVM < RS-NB	0.1027	0.9186	−3.0297	0.0037	−*3.1341*	*0.0027*	−1.7781	0.0807
Base versus Ensemble								
C4.5 < RS-C4.5	1.5089	0.1368	*3.4529*	*0.0011*	*3.4001*	*0.0012*	*4.9580*	*0.0000*
NN < RS-NN	1.4840	0.1432	*3.7333*	*0.0004*	*3.6663*	*0.0005*	*3.7754*	*0.0004*
SVM < RS-SVM	1.3702	0.1759	*3.5103*	*0.0009*	*3.5164*	*0.0009*	*6.2156*	*0.0000*
NB < RS-NB	1.4470	0.1533	*2.0191*	*0.0482*	*2.0188*	*0.0482*	*5.7430*	*0.0000*
(b) Segmenting type *s* = dislike								
Base versus Base								
C4.5 < NN	−0.0517	0.9590	−0.3484	0.7288	−0.3112	0.7568	1.3922	0.1692
C4.5 < SVM	−1.5922	0.1168	−0.3674	0.7146	−0.3674	0.7146	*2.2502*	*0.0283*
C4.5 < NB	−*2.1459*	*0.0361*	−*13.8448*	*0.0000*	−*13.8448*	*0.0000*	0.1839	0.8548
NN < SVM	−1.5282	0.1319	−0.0185	0.9853	−0.0555	0.9560	0.8677	0.3892
NN < NB	−*2.0790*	*0.0421*	−*13.5724*	*0.0000*	−13.5858	0.0000	−1.2081	0.2319
SVM < NB	−0.5914	0.5565	−*13.5946*	*0.0000*	−*13.5946*	*0.0000*	−*2.0673*	*0.0432*
Ensemble versus Ensemble								
RS-C4.5 < RS-NN	−0.4462	0.6571	0.8472	0.4004	1.3534	0.1812	*2.1927*	*0.0324*
RS-C4.5 < RS-SVM	−0.7100	0.4806	0.8186	0.4164	0.8827	0.3810	*2.5443*	*0.0137*
RS-C4.5 < RS-NB	−*12.1209*	*0.0000*	−*7.4664*	*0.0000*	−*7.4322*	*0.0000*	0.5340	0.5954
RS-NN < RS-SVM	−0.2651	0.7919	−0.0315	0.9750	−0.4770	0.6352	0.3537	0.7249
RS-NN < RS-NB	−*11.8060*	*0.0000*	−*8.2529*	*0.0000*	−*8.6805*	*0.0000*	−1.6677	0.1008
RS-SVM < RS-NB	−*11.5687*	*0.0000*	−8.2615	0.0000	−*8.2942*	*0.0000*	−*2.0211*	*0.0479*
Base versus Ensemble								
C4.5 < RS-C4.5	1.0205	0.3118	*2.2381*	*0.0291*	*2.2048*	*0.0315*	*4.0928*	*0.0001*
NN < RS-NN	0.6221	0.5363	*3.4212*	*0.0012*	*3.8486*	*0.0003*	*4.8828*	*0.0000*
SVM < RS-SVM	1.8912	0.0636	*3.4260*	*0.0011*	*3.4584*	*0.0010*	*4.3731*	*0.0001*
NB < RS-NB	−*9.2234*	*0.0000*	*9.7104*	*0.0000*	*9.7104*	*0.0000*	*4.4482*	*0.0000*
(c) Segmenting type *s* = sum								
Base versus Base								
C4.5 < NN	−*7.3255*	*0.0000*	−*4.9494*	*0.0000*	−*4.8977*	*0.0000*	−*4.0895*	*0.0001*
C4.5 < SVM	−*16.3176*	*0.0000*	−*9.1771*	*0.0000*	−*9.0680*	*0.0000*	0.2961	0.7682
C4.5 < NB	−*9.5597*	*0.0000*	−*8.9258*	*0.0000*	−*8.8171*	*0.0000*	−0.7126	0.4793
NN < SVM	−*7.9144*	*0.0000*	−*4.7452*	*0.0000*	−*4.8774*	*0.0000*	*4.3562*	*0.0001*
NN < NB	−0.7139	0.4790	−*3.8878*	*0.0004*	−*4.0311*	*0.0002*	*2.6493*	*0.0105*
SVM < NB	*10.5096*	*0.0000*	2.0088	0.0506	*2.0637*	*0.0450*	−0.9480	0.3476
Ensemble versus Ensemble								
RS-C4.5 < RS-NN	−0.3712	0.7119	−*12.3659*	*0.0000*	−*12.6411*	*0.0000*	−*24.3113*	*0.0000*
RS-C4.5 < RS-SVM	−*3.6321*	*0.0006*	−0.7347	0.4656	0.3079	0.7593	*8.8124*	*0.0000*
RS-C4.5 < RS-NB	−*8.3171*	*0.0000*	−*5.1562*	*0.0000*	−*4.8929*	*0.0000*	*10.1076*	*0.0000*
RS-NN < RS-SVM	−*3.2133*	*0.0022*	*10.4931*	*0.0000*	*11.6334*	*0.0000*	*27.1599*	*0.0000*
RS-NN < RS-NB	−*7.8203*	*0.0000*	*11.0239*	*0.0000*	*10.8831*	*0.0000*	*32.8773*	*0.0000*
RS-SVM < RS-NB	−*5.2629*	*0.0000*	−3.5703	0.0010	−*4.4613*	*0.0001*	−*2.0300*	*0.0485*
Base versus Ensemble								
C4.5 < RS-C4.5	−*2.1194*	*0.0384*	*3.9081*	*0.0003*	*3.4528*	*0.0012*	*16.1968*	*0.0000*
NN < RS-NN	*4.5425*	*0.0000*	−1.8326	0.0720	−1.6728	0.0998	−*8.4251*	*0.0000*
SVM < RS-SVM	*11.4120*	*0.0000*	*13.3717*	*0.0000*	*14.9017*	*0.0000*	*20.2885*	*0.0000*
NB < RS-NB	−*2.2939*	*0.0272*	*20.6640*	*0.0000*	*20.6640*	*0.0000*	*22.3312*	*0.0000*
(d) Segmenting type *s* = portfolio								
Base versus Base								
C4.5 < NN	−0.3357	0.7398	−1.2074	0.2385	−*3.2526*	*0.0028*	1.1173	0.2723
C4.5 < SVM	0.0746	0.9411	*2.4769*	*0.0197*	*2.5035*	*0.0185*	*3.4966*	*0.0016*
C4.5 < NB	−*2.7119*	*0.0115*	−*3.7995*	*0.0009*	−*3.8622*	*0.0008*	*6.9459*	*0.0000*
NN < SVM	0.5537	0.5836	*5.0627*	*0.0000*	*6.9119*	*0.0000*	*2.4446*	*0.0206*
NN < NB	−*3.2000*	*0.0031*	−*3.8374*	*0.0006*	−0.1194	0.9059	*6.1578*	*0.0000*
SVM < NB	−*3.7482*	*0.0007*	−*8.8516*	*0.0000*	−*8.9767*	*0.0000*	*4.2191*	*0.0002*
Ensemble versus Ensemble								
RS-C4.5 < RS-NN	−*5.9980*	*0.0000*	−*6.8700*	*0.0000*	−*6.9289*	*0.0000*	*9.1409*	*0.0000*
RS-C4.5 < RS-SVM	0.8009	0.4304	*4.4411*	*0.0001*	−0.4066	0.6870	*17.5249*	*0.0000*
RS-C4.5 < RS-NB	*2.7431*	*0.0110*	*4.0034*	*0.0004*	*2.8443*	*0.0080*	*12.4036*	*0.0000*
RS-NN < RS-SVM	*8.1948*	*0.0000*	*10.5347*	*0.0000*	*6.4963*	*0.0000*	*11.5459*	*0.0000*
RS-NN < RS-NB	*10.3702*	*0.0000*	*10.5375*	*0.0000*	*10.7623*	*0.0000*	*4.2133*	*0.0002*
RS-SVM < RS-NB	*2.6804*	*0.0115*	−0.8192	0.4189	*3.2946*	*0.0026*	−*7.8686*	*0.0000*
Base versus Ensemble								
C4.5 < RS-C4.5	1.8607	0.0720	*9.2073*	*0.0000*	*9.4357*	*0.0000*	*9.5237*	*0.0000*
NN < RS-NN	−*4.7197*	*0.0001*	*5.8578*	*0.0000*	*8.0650*	*0.0000*	*21.0127*	*0.0000*
SVM < RS-SVM	*4.0544*	*0.0003*	*12.7166*	*0.0000*	*8.4720*	*0.0000*	*34.1855*	*0.0000*
NB < RS-NB	*10.7037*	*0.0000*	*25.1731*	*0.0000*	*25.7502*	*0.0000*	*26.3625*	*0.0000*

The results are *t* and *p* values of the *t* tests for classification technique comparison, and the results more than 5 % of significance level are highlighted in italics

From Table 7, there are two key findings. First, there is no single classification technique that gave the best accuracy for all the feature sets in any given segmenting type. Second, ensemble learning methods are better than base learners in most of configurations. The reason is that the ensemble learning methods consider the writing style features in its entirety whereas the base learners only consider the average of the aggregated writing style features. This difference made the ensemble learning methods preserve the important information better than the base learners, and resulted in better accuracies.

### On comparisons of different ways in defining the classes of user reputations

In Table 4, regarding segmenting types, it is remarkable that the segmenting type *s* = dislike gave the highest precision for both Good and Bad classes. It means that segmenting users by their dislike scores is the best way in terms of precision. Moreover, the segmenting type s = portfolio gave the highest *F*-measure when the feature set F1 + F2 + F3 + F4 and RS-SVM are combined: 94.53 % for Good class and 94.47 % for Bad class. One possible reason is that the more accurately segmenting users by portfolio approach contributed to higher *F*-measure than the other segmenting types. However, because the segmenting type *s* = portfolio classifies users more strictly into Bad class, its bests in terms of precision and recall were worse than the bests of the other segmenting types.

Moreover, in Table 7, it is seen that, when *reputation*_portfolio_ was used, the feature set F1 + F2 + F3 + F4 and RS-SVM gave the best accuracy among all the configurations. The possible reason is that *reputation*_portfolio_ classified users into Good class if they are certainly good, and strictly filtered users into Bad class if we are unsure about whether they belong to Good or Bad class.

## Conclusions

This paper proposed a research framework to design and examine an automatic system that estimates user reputations of social media into Good and Bad classes by adopting writing styles. Using the most popular Web forum in South Korea, Daum Agora, selected as a test bed, the application was conducted by following the suggested research framework of the paper.

Consequently, the experimental results in Table 4 show that the configuration of the feature set F1 + F2 + F3 + F4 and RS-SVM gave the best accuracy, i.e. 94.50 %, when segmenting type *s* = portfolio. It proves possible to classify user reputations by writing style features in social media with high accuracy (*RQ1* is answered). In Table 6, the pairwise *t* tests on accuracy for different feature sets show that the feature set F1 + F2 + F3 + F4 ranked the best for all eight classification techniques regardless of segmenting types (*RQ2* is answered). It represents that keywords and phrases on certain topics affect user reputations more than the other content-free writing style features. Whereas, according to Table 7, the results of pairwise *t* tests on accuracy for different classification techniques show that there was no single classification technique that gave the best accuracy for all the feature sets in any given segmenting type, but ensemble learning methods turned out better than base learners (*RQ3* is answered). The experimental results related to *RQ2* and *RQ3* indicate that both the feature set F1 + F2 + F3 + F4 and the ensemble learning method are respectively better for handling with a large set of writing style features, and such common strength provided a synergy effect. In addition, the paper concluded that combining two types of online user feedbacks by using portfolio approach, i.e. segmenting type *s* = portfolio, gave the better accuracy than the other segmenting types (*RQ4* is answered). A potential explanation is that, because the suggested portfolio approach segments user reputations more strictly into Good and Bad classes, it is better able to address the problem of this study.

This paper contributes to the literature review as follows. First, this study is the first work that adopts writing styles as objective features to automatically classify social media user reputations into Good and Bad classes. Second, this paper provided guidelines for the system implementation in two ways: (1) which writing style features and classification technique should be used together for the best accuracy; (2) which segmenting type gave the best result with respect to accuracy. In particular, because social media have similar ways in measuring user reputations, which are given as the online user feedbacks, e.g. like, dislike, or both of two, the results can be used as a reference for similar studies on the other types of social media. Third, the paper helps keep the healthy and trustful social media ecosystem by protecting users from bad users, and it enables us to manage user reputations that are manipulated to be either lower or higher than the original values. As a consequence, it helps build the trust between users by complementing the online user feedback system in social media.

Directions for further studies can be suggested based on this paper as follows. First, for this study, South Korea was selected as the test bed country for reasons, but different country targets and more various languages may lead to additional implications. Hence, future researches for various countries, e.g. US, European, China, Japan, and Mid East are recommendable as the future researches. Second, this study focused on writing styles as objective features to classify user reputations in social media, but there can be other objective features useful for this study, e.g. network structures in communications between users and their commenters. In a similar vein, third, simpler or more sophisticated approaches should be considered to tackle the computing problem that ensemble learning methods take a great deal of time. Thus, the further studies can be conducted to revisit the problems and challenging issues, which motivated this study, with different perspectives on countries, languages, features, and techniques.
